# Bacterial Genotoxins Promote Inside-Out Integrin β1 Activation, Formation of Focal Adhesion Complexes and Cell Spreading

**DOI:** 10.1371/journal.pone.0124119

**Published:** 2015-04-13

**Authors:** Laura Levi, Tatsushi Toyooka, Manuel Patarroyo, Teresa Frisan

**Affiliations:** 1 Department of Cell and Molecular Biology, Karolinska Institutet, Stockholm, Sweden; 2 Department of Dental Medicine, Karolinska Institutet, Stockholm, Sweden; Thomas Jefferson University, UNITED STATES

## Abstract

Integrins are membrane bound receptors that regulate several cellular processes, such as cell adhesion, migration, survival and proliferation, and may contribute to tumor initiation/progression in cells exposed to genotoxic stress. The extent of integrin activation and its role in cell survival upon intoxication with bacterial genotoxins are still poorly characterized. These toxins induce DNA strand breaks in the target cells and activate the DNA damage response (DDR), coordinated by the Ataxia Telangectasia Mutated (ATM) kinase. In the present study, we demonstrate that induction of DNA damage by two bacterial genotoxins promotes activation of integrin β1, leading to enhanced assembly of focal adhesions and cell spreading on fibronectin, but not on vitronectin. This phenotype is mediated by an ATM-dependent inside-out integrin signaling, and requires the actin cytoskeleton remodeler NET1. The toxin-mediated cell spreading and anchorage-independent survival further relies on ALIX and TSG101, two components of the endosomal sorting complex required for transport (ESCRT), known to regulate integrin intracellular trafficking. These data reveal a novel aspect of the cellular response to bacterial genotoxins, and provide new tools to understand the carcinogenic potential of these effectors in the context of chronic intoxication and infection.

## Introduction

Bacterial genotoxins are a novel group of toxins that induce DNA damage into the target cell. At present only three bacterial genotoxins have been identified. Two are protein toxins: the cytolethal distending toxin (CDT) family produced by a number of Gram-negative bacteria and the typhoid toxin produced by *Salmonella enterica* serovar Typhi (reviewed in [1]). The third member, colibactin, is a peptide-polyketide genotoxin, produced by strains belonging to the phylogenetic group B2 of *Escherichia coli* (reviewed in [2]).

CDTs are produced from three linked genes, which are designated *cdtA*, *cdtB* and *cdtC* and encode the CdtA, CdtB, CdtC proteins. The CdtB subunit is functional and structural homologous to mammalian DNase I [3–5]. The CdtA and CdtC accessory subunits are required for the toxin binding and possibly for the proper intracellular trafficking of the active subunit to the nucleus, where it exerts its genotoxic activity (reviewed [1]). Intoxication with CDT promotes the formation of DNA breaks in target cells [6–8], and activates the classical DNA damage response (DDR) orchestrated by the phosphatidylinositol 3-kinase-like protein kinase ataxia telangiectasia-mutated (ATM) [9–15,16]. As consequence of the DDR activation cells are arrested in the G1 and/or G2 phases of the cell cycle. Failure to repair the damage induces senescence or apoptosis in a cell type-dependent manner (reviewed in [1,17]). However, intoxicated cells occasionally survive and overcome the DDR-induced cell death or cellular senescence, leading to the acquisition of genomic instability and the capacity to grow in an anchorage independent manner [18].

Few studies have addressed the activation of survival signals in cells exposed to bacterial genotoxins. In adherent cells, CDT intoxication is associated with formation of actin stress fibers [10,19], via activation of the small GTPase RhoA, leading to survival of the intoxicated cells [6,20]. RhoA activation and cell survival are coordinated by the ATM-dependent DNA damage response [6], which requires the functional RhoA-specific guanine nucleotide exchange factor (GEF) NET1 [20].

Stress fibers are usually anchored at focal adhesion complexes that form upon engagement of integrins with components of the extracellular matrix (ECM) (reviewed in [21]). Integrins are αβ heterodimeric receptors, comprising 8 β and 18 α subunits in mammals, which can assemble into 24 distinct integrins and transduce bi-directionally across the plasma membrane cues from the surrounding microenvironment [22,23]. Integrins can transduce signals in two ways: i) the outside-in signalling pathway by binding of the extracellular domain to components of the ECM; ii) the inside-out signalling pathway, where integrins switch from a low to a high affinity state via activation of other surface molecules, such as cytokines receptors or growth factors receptors (reviewed in [22,23]).

Integrins regulate many intracellular processes, several of them associated with acquisition of hallmarks of cancer, such as cell survival and proliferation, prevention of anoikis, promotion of cell migration, and angiogenesis [24,25]. Based on this evidence, we have assessed whether cellular exposure to bacterial genotoxins promotes integrin activation.

We demonstrate that the toxin-induced DNA damage leads to an ATM-dependent activation of integrin β1, resulting in enhanced cell spreading on fibronectin, and increased formation of focal adhesion complexes. Blocking antibodies directed against integrin β1 and α5 chains inhibit this phenotype. The toxin-induced cell spreading is dependent on the guanine exchange factor NET1, a cytoskeleton re-modeller, and ALIX and TSG101, two components of the endosomal sorting complex required for transport (ESCRT) [26].

These data reveal a novel aspect of the DNA damage response to bacterial genotoxins, namely the ATM-dependent inside-out signaling activation of integrin β1, and provide new insights to understand the carcinogenic properties of CDT [18,27].

## Material and Methods

### Cell culture and media

HeLa, U2OS, and CaCo2 cell lines were purchased from ATCC (LGC Standards Teddington, Middlesex, UK) and maintained in Iscove’s Modified Dulbecco’s Medium (IMDM, Sigma-Aldrich, St. Louis, MO, USA) containing 2 mM L-Glutamine (Sigma-Aldrich), 10% fetal bovine serum (FBS) (Invitrogen, Grand Island, NY, USA), and 10μg/ml Ciprofloxacin (Sigma-Aldrich) (complete medium) at 37°C in a humid atmosphere of 5% CO_2_.

Intoxication was performed by incubating the cells for the indicated periods of time with the *Haemophilus ducreyi* CDT (1μg/ml) in complete medium. Production and purification of the *H*. *ducreyi* CDT subunits were previously described [6,28].

The *S*. *enterica* serovar Typhimurium strain MC71 expressing a functional typhoid toxin (MC71TT), and the isogenic strain, carrying a non-functional toxin due to a deletion of the TT active subunit (MC71Δ*cdtB*), were previously described [29]. Bacteria were grown in Luria-Bertani (LB) medium, supplemented with 50μg/ml kanamycin. HeLa and U2OS cell lines were infected performing a gentamicin protection assay as previously described [30] at a multiplicity of infection of 50:1.

### Cell adhesion and spreading assay

Four hundred thousand cells/well were seeded in a 6-well plate and intoxicated with CDT (1μg/ml) for the indicated periods of time 24h after plating. To assess adhesion, cells were detached with EDTA 2.5 mM in phosphate buffered saline (PBS) and seeded at the concentration of 1x10^5^ cells/well on 13 mm cover glass pre-coated with 1 μg/ml fibronectin (Sigma-Aldrich) or 1 μg/ml vitronectin (R&D Systems, Minneapolis, MN, USA) in PBS, and let adhere for 20 minutes in complete medium. The slides were then washed once in PBS, fixed with 4% paraformaldehyde for 10 minutes, and immunofluorescence analysis was performed as described below.

When indicated, cells were incubated in the presence of ATM inhibitor KU-55933 5μM (Millipore, Billerica, MA, USA) for 1h prior intoxication. Cells were further intoxicated for the indicated periods of time in the presence of the ATM inhibitor.

For the antibody blocking experiments, three millions cells were seeded in a 10 cm diameter dish. The following day, cells were left untreated or intoxicated with CDT (1μg/ml), detached with EDTA 2.5 mM in PBS, counted, and incubated (1.5x10^5^ cells) with the indicated function-blocking antibody (final concentration 10μg/ml) in 200μl of PBS for 30 minutes at room temperature. Normal mouse (NMS) or rat (NRS) serum was used as negative control. Cells were seeded on fibronectin-coated glasses for 20 minutes in 2 ml complete medium, fixed with 4% paraformaldehyde for 10 minutes, and immunofluorescence analysis was performed as described below. The following function-blocking antibodies were used: anti-integrin β1 monoclonal antibody 13 (rat IgG, BD Bioscience, San Jose, CA, USA), and anti-integrin α5 monoclonal antibody P1D6 (mouse IgG, Millipore Billerica, MA, USA).

### Immunofluorescence

After fixation in 4% paraformaldehyde, slides were washed twice in PBS, and further incubated with 0.2% Triton X-100, 3% BSA in PBS for 30 minutes at room temperature for blocking and permeabilization. Paxillin was visualized using the mouse monoclonal antibody clone 5H11 purchased from Millipore (dilution 1:100 in PBS) for 1h at room temperature, followed by the anti-mouse FITC-conjugated secondary antibody (DAKO, Glostrup-Denmark, dilution 1:100 in PBS) for 1h at room temperature. The actin cytoskeleton was visualized by staining with TRITC-phalloidin, as previously described [6]. Nuclei were counterstained with DAPI (Vector Laboratories Inc, Burlingame, CA, USA). Slides were mounted and viewed using a Leica DMI 6000 B, equipped with Hamamatsu ORCA-R2 digital camera. Cell area was measured using ImageJ software (http://rsbweb.nih.gov/ij/).

### RNA interference and transfections

A total of 1x10^5^ HeLa cells were seeded in 12-well plates in 1 ml of complete medium. Transfection was performed using the forward protocol with the INTERFERin reagent purchased from Polyplus Transfection (Berkeley, CA, USA), according to the manufacturer’s instructions. Gene silencing was assessed by western blot analysis 48h after transfection. The following duplex small interfering RNAs (siRNAs) were used: human NET1 siRNA (esiNET1) purchased from Sigma-Aldrich, human TSG101 siRNAs (s14440 and s14441) purchased from Ambion (Life technologies, Carlsbad, CA, USA), human ALIX siRNA (Hs_PDCD6IP_5 and Hs_PDCD6IP_8), and Allstars Negative Control siRNA #102780 (scRNA) purchased from Qiagen (Hilden, Germany).

### Anchorage independent cell survival

Cells were left untreated or intoxicated for 6h with CDT (1μg/ml), detached with 2.5 mM EDTA in PBS, and re-seeded at the concentration of 1x10^5^ cells/well in a 12-well plate, pre-coated with 1% agarose-TrisHCl 10 mM pH 7.4 to prevent adhesion on the bottom of the well. Cell number and viability was assessed every 24h starting from 48h after seeding using Trypan blue solution 0.4% (Sigma-Aldrich).

### FACS analysis

Two millions HeLa cells were seeded in a 10 cm diameter dish, left untreated, irradiated (8 Gy), or intoxicated with CDT (1μg/ml) for the indicated periods of time. Cells were then detached with 2.5 mM EDTA in PBS. Four hundred thousand cells were incubated with the primary antibody (diluted 1:50 in PBS) on ice for 45 minutes. As positive control for integrin activation, cells were incubated with the primary antibody in PBS without Ca^2+^ and Mg^2+^ containing 2 mM MnCl_2_ as previously described [31]. As negative control, cells were incubated with isotype-matched immunoglobulins. After washing in PBS, cells were incubated with the Alexa Fluor 555 donkey anti-mouse secondary antibody (Life technologies) for 45 minutes on ice. Flow cytometry analysis was performed using a FACSCalibur (BD Bioscience, San Jose, CA, USA). Data from 1x10^4^ cells were collected and analyzed using the CellQuest Pro software (BD Bioscience).

The following monoclonal antibodies were used: the pan anti-integrin β1 4B7R (R&D Systems) and HUTS-21 (BD Bioscience) that recognizes the activated form of integrin β1 [31].

### Western blot analysis

Proteins were fractionated by SDS-polyacrylamide gel electrophoresis using precast 4–12% gradient gels (Invitrogen), transferred to polyvinylidene difluoride (PVDF) membranes (Millipore) and probed with 1:1000 dilution of the indicated antibodies, followed by the appropriate horseradish peroxidase-conjugated secondary antibody (GE Healthcare, Piscataway, NJ, USA). The blots were developed by enhanced chemiluminescence (GE Healthcare) according to the instructions of the manufacturer. The following antibodies were used: goat polyclonal antibody against NET1 (Abcam, Cambridge, UK), rabbit polyclonal antibody against TSG101 and mouse monoclonal antibody against β-actin (clone A5541, Sigma-Aldrich), mouse monoclonal antibody against ALIX (clone 3A9), and rabbit polyclonal antibodies anti-phospho-CHK2 and H2AX (Cell Signaling, Beverly, MA, USA).

## Results

### Bacterial genotoxins promote inside-out integrin β1 activation

**Fig 1:**
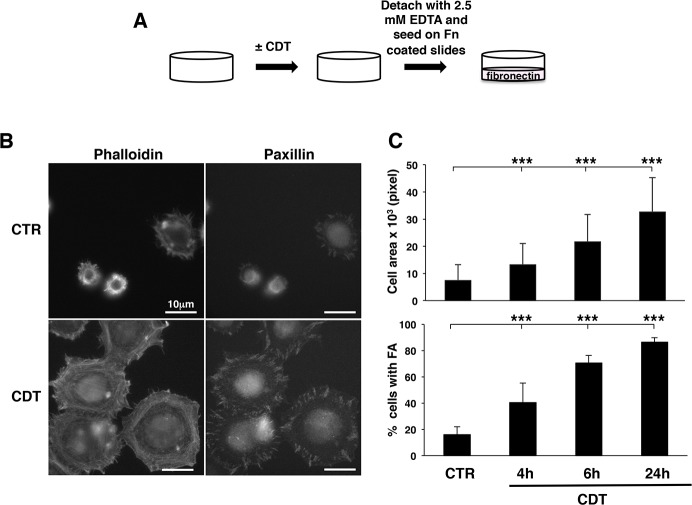
CDT intoxication promotes focal adhesions and cell spreading. **A.** Protocol for the adhesion/spreading assay. HeLa cells were seeded and further left untreated or intoxicated with CDT (1μg/ml) for 4, 6 or 24h. At the indicated time points, cells were detached with 2.5 mM EDTA in PBS, and seeded on fibronectin (Fn)-coated glasses for 20 minutes, as described in Material and Methods, prior fixation and immunofluorescence analysis. **B.** Representative micrographs of HeLa cells untreated (CTR) or intoxicated for 24h (CDT) after 20 minutes adhesion on fibronectin-coated glasses. The actin cytoskeleton was visualized by phalloidin staining (left panel) and the focal adhesion complexes were visualized using an anti-paxillin antibody (right panel). **C.** Quantification of cell spreading and formation of focal adhesion complexes of HeLa cells left untreated (CTR) or intoxicated (CDT) for the indicated period of times. The upper panel shows the area of 400 randomly selected adherent cells, measured using the ImageJ software. The lower panel shows the percentage of cells carrying more than 5 focal adhesion complexes/cell, identified by paxillin staining. The data are presented as the mean ± SEM of three independent experiments. Statistical analysis was performed using the Student t-test. *** p value < 0.001.

We have previously demonstrated that exposure to the genotoxin CDT promotes RhoA-dependent formation of actin stress fibers in cells of epithelial and mesenchymal origin [6]. Since stress fibers formation is intimately linked to integrin activation (reviewed in [32]), we asked whether exposure to bacterial genotoxins promotes activation of the integrin signaling. To this end, we have designed a series of experiments where CDT-treated HeLa cells were detached with 2.5 mM EDTA to preserve the integrin structure on the cell surface, and seeded on fibronectin-coated coverslips for 20 minutes (Fig 1A). Activation of integrin signaling was investigated by measuring the capacity of cells to spread, assessed by staining of the cellular cytoskeleton with TRITC-labelled phalloidin, and to form focal adhesion (FA) complexes, assessed by immunofluorescence analysis of paxillin [33]. As shown by a representative experiment shown in Fig 1B, and the quantification of three independent experiments shown in Fig 1C, time-dependent intoxication was associated with increased cell spreading and promotion of paxillin-positive FA. This effect was already detected 4h post-intoxication and increased 24h after toxin exposure. The phalloidin staining revealed that, upon intoxication and promotion of cell spreading, the cells presented a re-organization of the actin cytoskeleton into a peripheral ring of polymerized actin as well as transversal stress fibers (Fig 1B).

**Fig 2:**
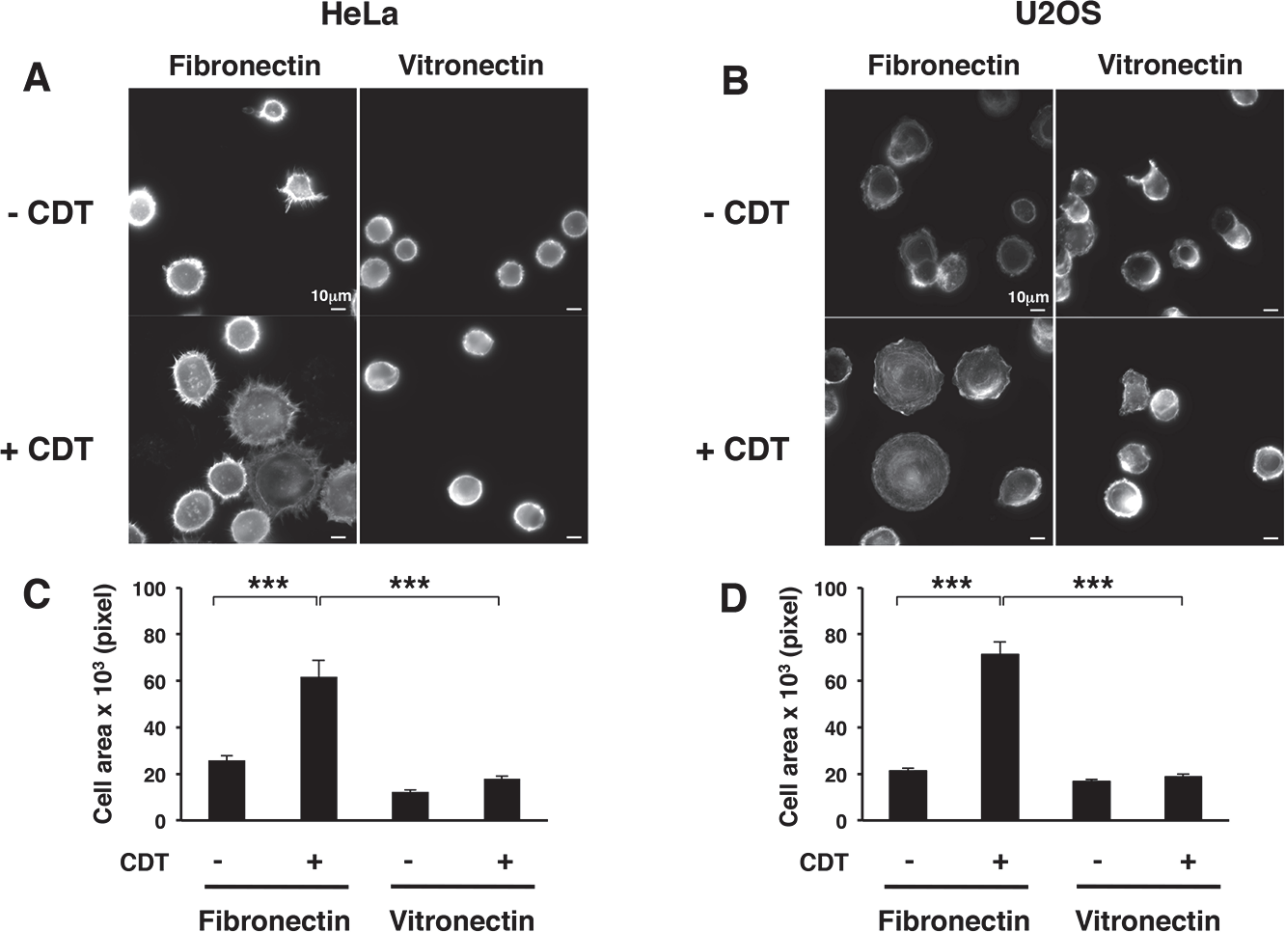
CDT intoxication promotes cell spreading on fibronectin but not on vitronectin. HeLa (**A**) or U2OS (**B**) cells, left untreated (- CDT) or intoxicated with CDT (1μg/ml) for 6h (+ CDT), were detached with 2.5 mM EDTA in PBS, seeded on fibronectin- or vitronectin-coated glasses for 20 minutes, prior fixation and immunofluorescence analysis. The actin cytoskeleton was visualized by phalloidin staining. Quantification of cell spreading in HeLa (**C**) and U2OS (**D**) cells, as described in Fig 1. The graph shows the area of 100 randomly selected adherent cells, measured using the ImageJ software. The data are presented as the mean ± SEM. Statistical analysis was performed using the Student t-test. *** p value < 0.001.

To assess whether intoxication induces activation of cell-surface receptors with different substrate specificity, we investigated the extent of adhesion on vitronectin-coated coverslips. CDT treatment did not promote enhanced adhesion of HeLa and U2OS cells on this ligand (Fig 2A–2D).

**Fig 3:**
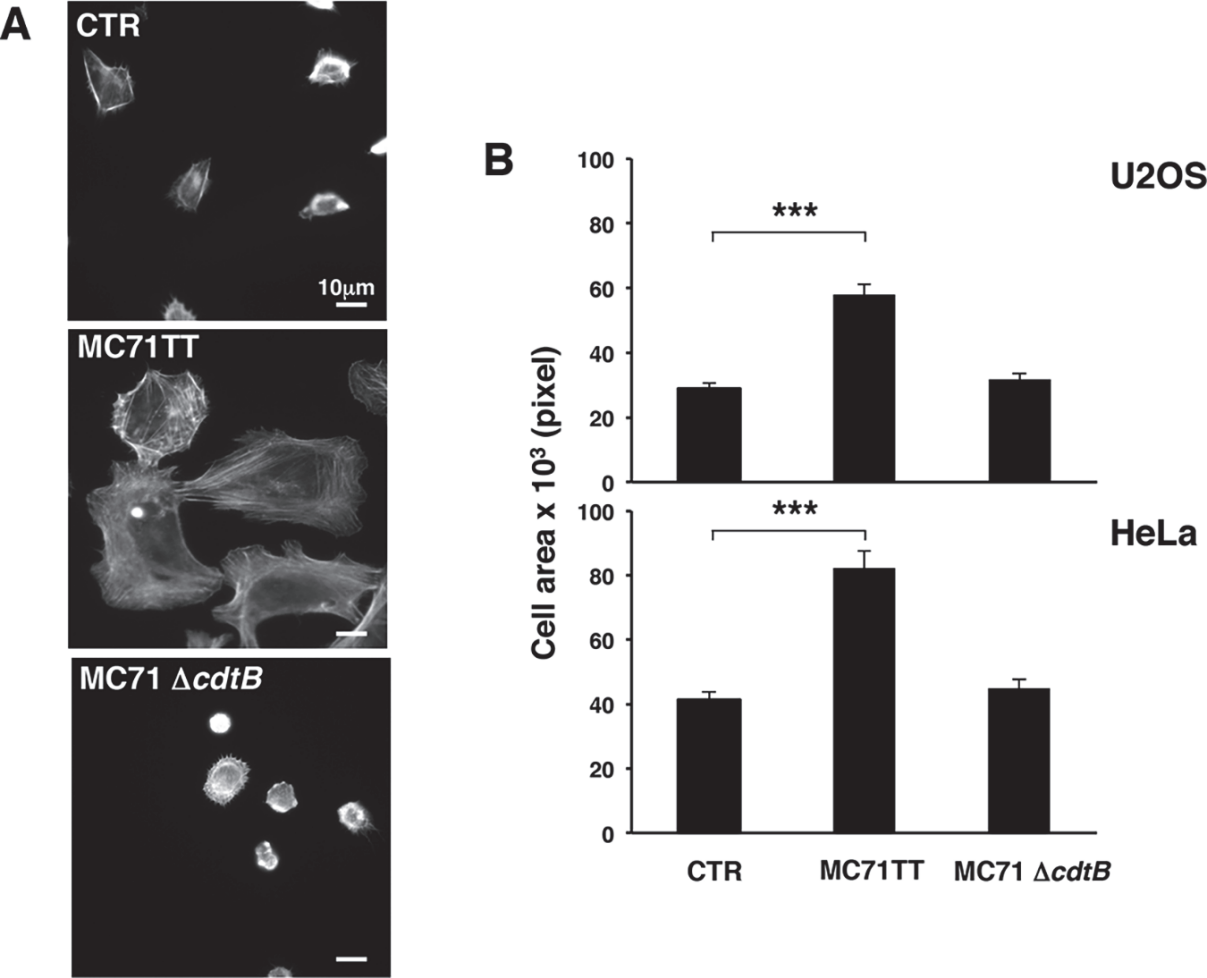
Infection with toxingenic *S*. *typhimurium* promotes cell spreading. **A.** U2OS cells were left untreated or infected for 24h with the *S*. *typhimurium* strains carrying the active typhoid toxin (MC71TT) or an isogenic strain carrying a deletion mutant of the active subunit, thus lacking the genotoxic activity (MC71Δ*cdtB*). Cells were detached with 2.5 mM EDTA in PBS, seeded on fibronectin-coated glasses for 20 minutes, prior fixation and immunofluorescence analysis. The actin cytoskeleton was visualized by phalloidin staining. **B.** Quantification of cell spreading of U2OS or HeLa cells left untreated or infected, as described in Fig 1. The area of 50 adherent cells per experiment was measured using the ImageJ software. The data are presented as the mean ± SEM of three independent experiments. Statistical analysis was performed using the Student t-test. *** p value < 0.001.

Enhanced cell spreading on fibronectin was not limited to cells exposed to the soluble CDT, but was also detected in HeLa and U2OS cells infected for 24h with the *Salmonella typhimurium* strain MC71, expressing the genotoxin known as typhoid toxin (MC71TT) [29]. This effect was not observed in control cells or cells infected with an isogenic strain carrying a deletion of the gene encoding for the active toxin subunit (MC71Δ*cdtB*), thus unable to induce DNA damage (Fig 3A and 3B).

These data suggest that activation of the integrin signaling is a general response of epithelial and mesenchymal cells to bacterial genotoxins.

Since we were interested in assessing the early events that promoted cell spreading and FA formation, avoiding confounding effects associated with 24h intoxication, we performed the subsequent experiments using cells exposed to CDT for 6h, unless otherwise specified.

**Fig 4:**
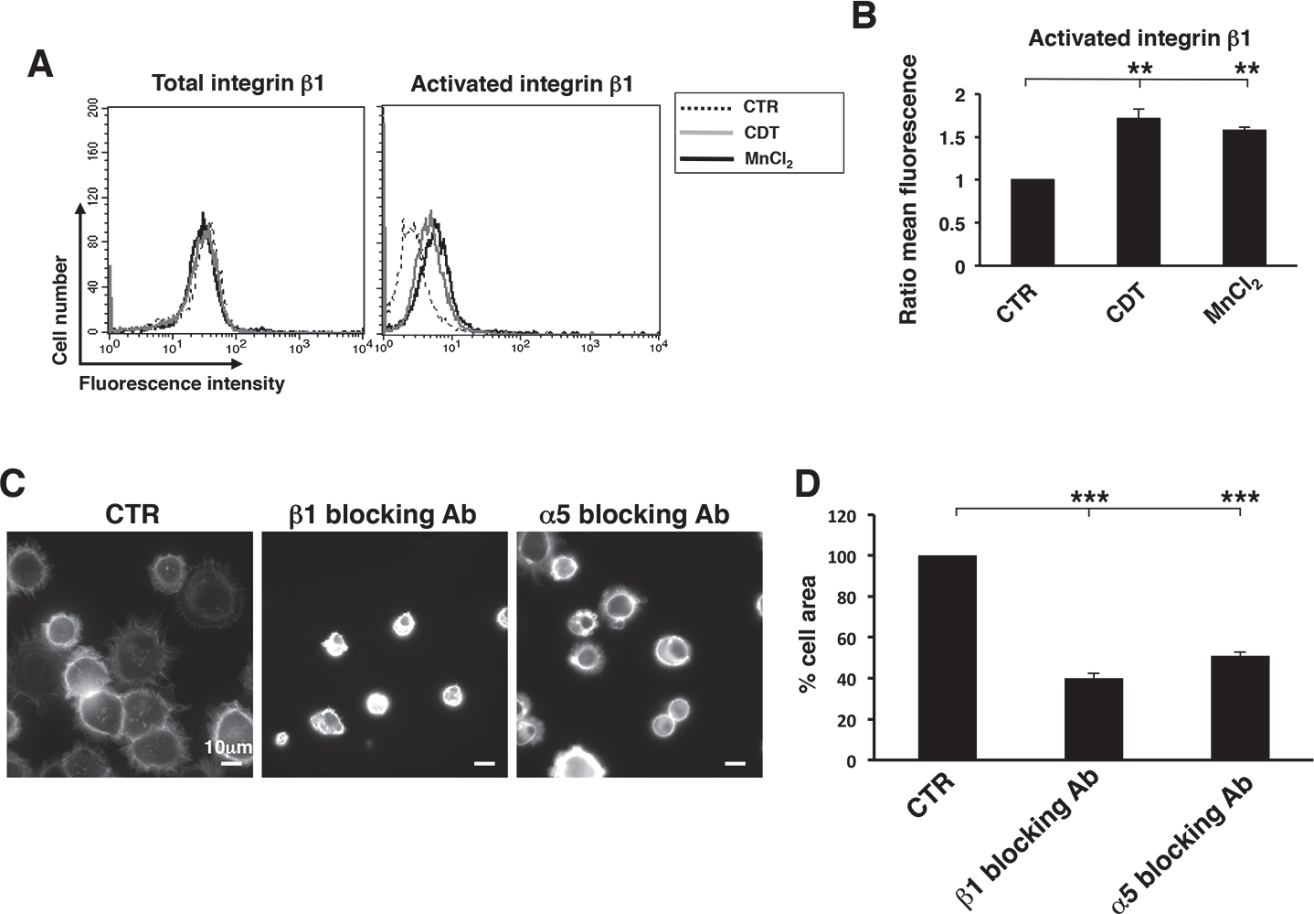
CDT intoxication promotes activation of β1 integrin. **A.** HeLa cells were seeded, and further left untreated (dotted line), intoxicated with CDT (1μg/ml, grey line) for 6h, or treated with 2 mM MnCl_2_ as described in Material and Methods (black line). Expression of the integrin β1 total levels (left panel) or its activate form (right panel) was assessed by FACS analysis. **B.** Quantification of the activated form of integrin β1. The data are presented as ratio between the mean fluorescence intensity of the intoxicated or MnCl_2_-treated cells and the mean fluorescence intensity of the untreated cells. Mean ± SEM of 5 independent experiments. Statistical analysis was performed using the Student t-test. ** p value < 0.01. **C.** Cell adhesion/spreading assay was performed on HeLa cells left untreated or intoxicated with CDT for 6h prior detachment with 2.5 mM EDTA in PBS. Before adhesion on fibronectin-coated glasses, cells were incubated for 30 minutes with the rat monoclonal antibody 13 directed against integrin β1 or mouse monoclonal antibody P1D6 directed against integrin α5. As control, cells were incubated with normal rat or mouse serum (CTR). The figure shows representative micrographs where the actin cytoskeleton is visualized by phalloidin staining. **D.** Quantification of the cell spread was performed as described in Fig 1. Data are presented as percentage of the cell area relative to the cells pre-incubated with the normal rat or mouse serum. Mean ± SEM of three independent experiments. Statistical analysis was performed using the Student t-test. *** p value < 0.001.

The enhanced formation of FA observed upon intoxication, suggested that toxin-induced DNA damage promoted activation of integrins. To investigate this issue, HeLa cells were intoxicated for 6h and subsequently detached with 2.5 mM EDTA. The surface levels of integrin β1 and its activated form were assessed by indirect immunofluorescence followed by FACS analysis. Intoxication promoted a significant increase of the activated form in absence of an up-regulation of the total levels of surface expression (Fig 4A and 4B). The levels of integrin activation in intoxicated cells were comparable to those observed in cells treated with 2 mM MnCl_2_, a known activator of these surface receptors [31]. A similar extent of integrin β1 activation was promoted in cells exposed to another genotoxic stress, namely ionizing radiation (IR) (S1 Fig). These data indicate that DNA damage, caused either by the toxin or IR, triggers an inside-out signaling that results in a conformational change of the integrin β1, allowing a higher affinity binding with its ligand [34]. We cannot exclude that intoxication also increases the integrin density on plasma membrane microdomains, thus modulating the avidity of the binding [34]. However, we did not detect any significant re-localization of integrin molecules on the cell surface by confocal microscopy analysis (data not shown).

The role of integrin on the CDT-induced phenotype was confirmed by a 50% decrease in the cell area, when HeLa cells were pre-incubated with blocking antibodies against the integrin β1 or α5 subunits, prior seeding onto fibronectin-coated coverslips (Fig 4C and 4D).

### The ATM kinase is required for the toxin-induced integrin β1 activation

As a next step, we asked whether integrin activation was dependent on the DNA damage response induced by the intoxication. To this end, we used a specific inhibitor of the ATM kinase, one of the key proteins that senses DNA double strand breaks and orchestrates the DNA repair and checkpoint responses [35]. To define the amount of inhibitor that was sufficient to block the ATM-dependent response, we performed titration experiments where HeLa cells were pre-incubated with the indicated concentration of the ATM inhibitor KU-55933 for 1h, prior exposure to CDT for additional 6h. Non-intoxicated cells pre-treated with the same inhibitor concentrations were used as control. Phosphorylation of two ATM effectors, CHK2 and H2AX, was used to monitor activation of the ATM-dependent DDR by western-blot analysis (S2 Fig). Pre-incubation of cells with KU-55933 at the concentration of 5μM was sufficient to decrease significantly the CDT-induced phosphorylation of both CHK2 and H2AX. Based on these results, we selected this concentration for the next series of experiments.

**Fig 5:**
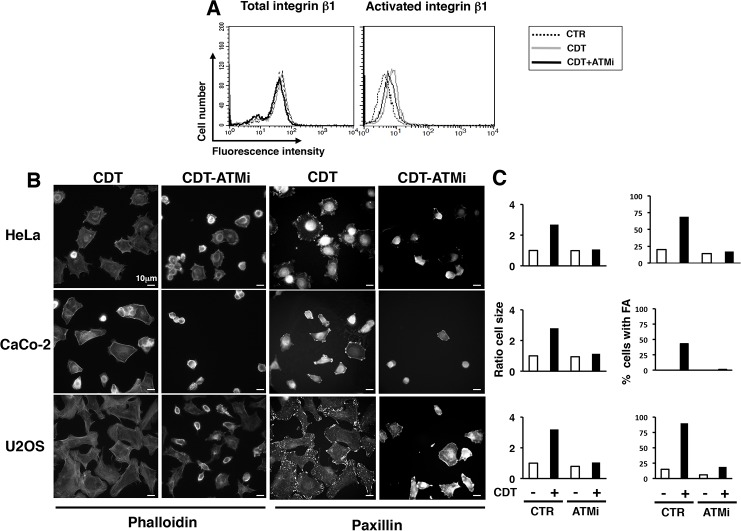
Integrin β1 activation and enhanced spreading in intoxicated cells is dependent on the DNA damage sensor kinase ATM. **A.** HeLa cells were left untreated (CTR, dotted line), intoxicated (1μg/ml for 24h, CDT, grey line) or pre-incubated with the ATM inhibitor KU-55933 (5μM, ATMi, black line) prior intoxication. Surface expression of the integrin β1 total levels (left panel) or its activate form (right panel) was assessed by FACS analysis. **B.** Analysis of cell spreading and formation of focal adhesions was performed in HeLa, CaCo2, and U2OS cells, pre-incubated with the ATM inhibitor as described in A. The panel shows representative micrographs of intoxicated cells left untreated (CDT) or pre-incubated with the ATM inhibitor (CDT-ATMi). The actin cytoskeleton was visualized by phalloidin staining and focal adhesion complexes were visualized using an anti-paxillin antibody. **C.** Quantification of cell spreading and formation of focal adhesion complexes was performed as described in Fig 1. The cell area is presented as ratio between the mean of the cell area in intoxicated cells and the mean of the cell area in non-intoxicated cells.

Pre-incubation of HeLa cells with 5μM KU-55933 prior intoxication reduced the levels of integrin β1 activation of approximately 50% (Fig 5A). Next, we assessed the effect of the ATM inhibitor on cell spreading and formation of FA in HeLa, Caco-2, and U2OS cells seeded onto fibronectin-coated coverslips. As shown in Fig 5B and 5C, CDT-induced DNA damage promoted a 3-fold increase in cell spreading relative to the non-intoxicated cells, as assessed by phalloidin staining. This was associated with a 5- to 8-fold increase in the number of cells positive for FA complexes, as assessed by paxillin immunofluorescence analysis. These effects were completely abolished when ATM was inhibited prior intoxication in all the cell lines tested, indicating that integrin activation, cell spreading and formation of FA are direct consequences of the ATM-dependent DNA damage response. Since the ATM inhibitor only partially affected integrin activation (Fig 5A), it is likely that this kinase regulates additional pathway(s) that mediate adhesion and spreading in intoxicated cells.

**Fig 6:**
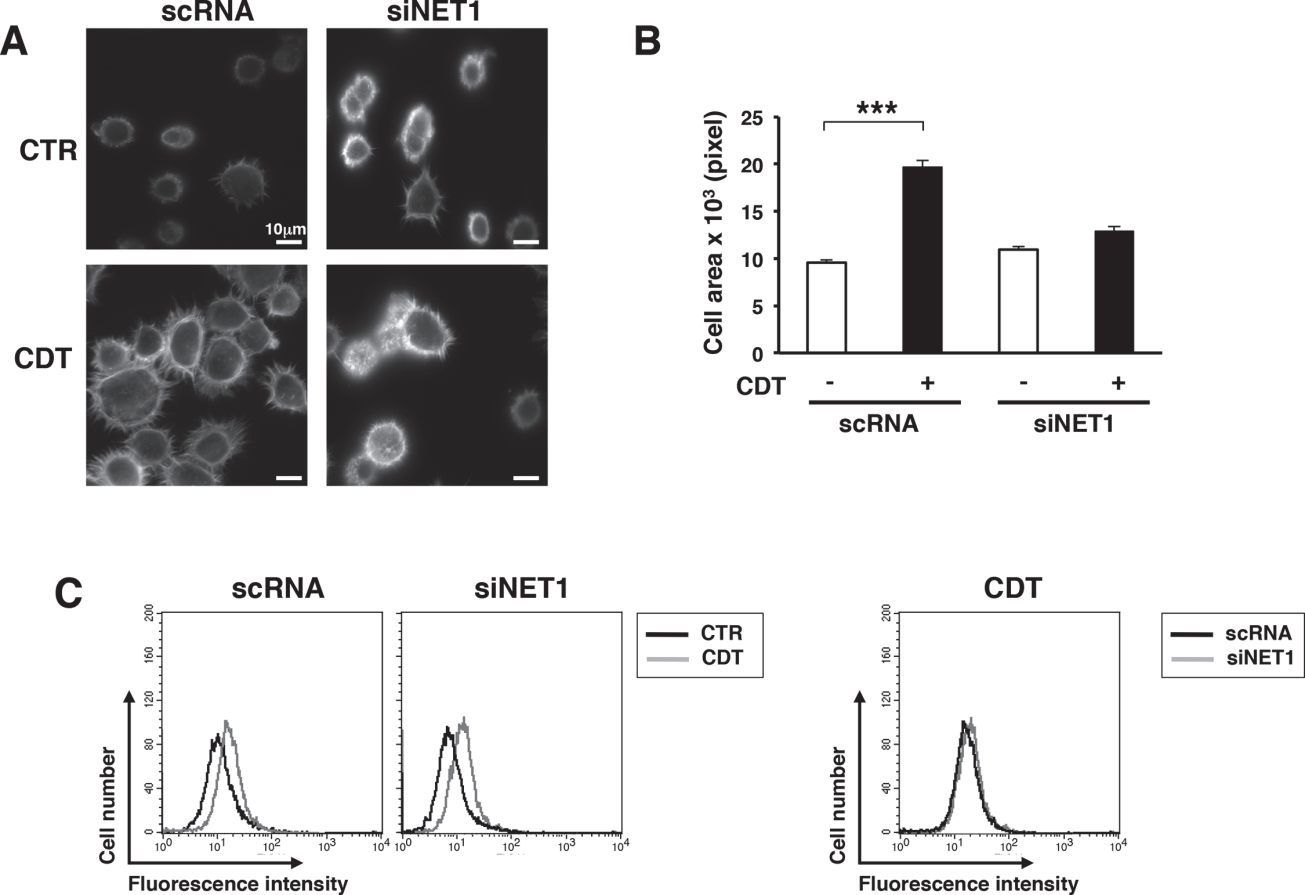
NET1 is required to promote cell spreading upon CDT intoxication. **A.** HeLa cells, transfected with the non-silencing siRNA (scRNA) or the NET1 specific siRNA (siNET1), were left untreated (CTR) or intoxicated with CDT (1μg/ml) for 6h, prior adhesion for 20 minutes on fibronectin-coated glasses. The figure shows representative micrographs where the actin cytoskeleton was visualized by phalloidin staining. **B.** The quantification of the cell area was performed as described in Fig 1. The data are presented as the mean ± SEM of three independent experiments. Statistical analysis was performed using the Student t-test. *** p value < 0.001 **C.** HeLa cells, transfected with the indicated siRNA, were left untreated (CTR) or intoxicated (1μg/ml for 24h, CDT). Surface expression of the activated β1 integrin was assessed by FACS analysis (left panel). The right panel shows the overlay of the mean fluorescence intensity in CDT-treated cells expressing normal (scRNA) or decreased (siNET1) levels of NET1.

We have previously shown that ATM modulates the cellular response to the CDT-induced DNA damage not only in the nucleus, activating the DNA repair and checkpoint responses [12,13], but also in the cytosol, leading to activation of the small GTPase RhoA, via the GEF NET1 [20], leading to a pronounced re-organization of the actin cytoskeleton. Therefore, we asked whether NET1 was also a key effector in transducing the DNA-damage-dependent signaling leading to cell spreading and adhesion. The endogenous levels of NET1 were down-regulated by siRNA. HeLa cells transfected with a non-silencing siRNA (scRNA) were used as control. Expression of NET1 was decreased by approximately 80% 48h after transfection compared to the levels observed in cells transfected with the control scRNA (S3A Fig, and [20]). As expected, CDT intoxication resulted in a two-fold increase in the spreading capacity of control cells upon seeding on fibronectin-coated glasses, while this effect was completely abolished upon NET1 knock-down (Fig 6A and 6B). However, the levels of activated integrin β1 were not altered upon NET1 down-regulation (Fig 6C), suggesting that this effector regulates events downstream of the interaction of the integrin β1 with the ECM [36].

### The ESCRT proteins ALIX and TSG101 are required for the CDT-induced integrin β1 signaling and adhesion-independent cell survival

**Fig 7:**
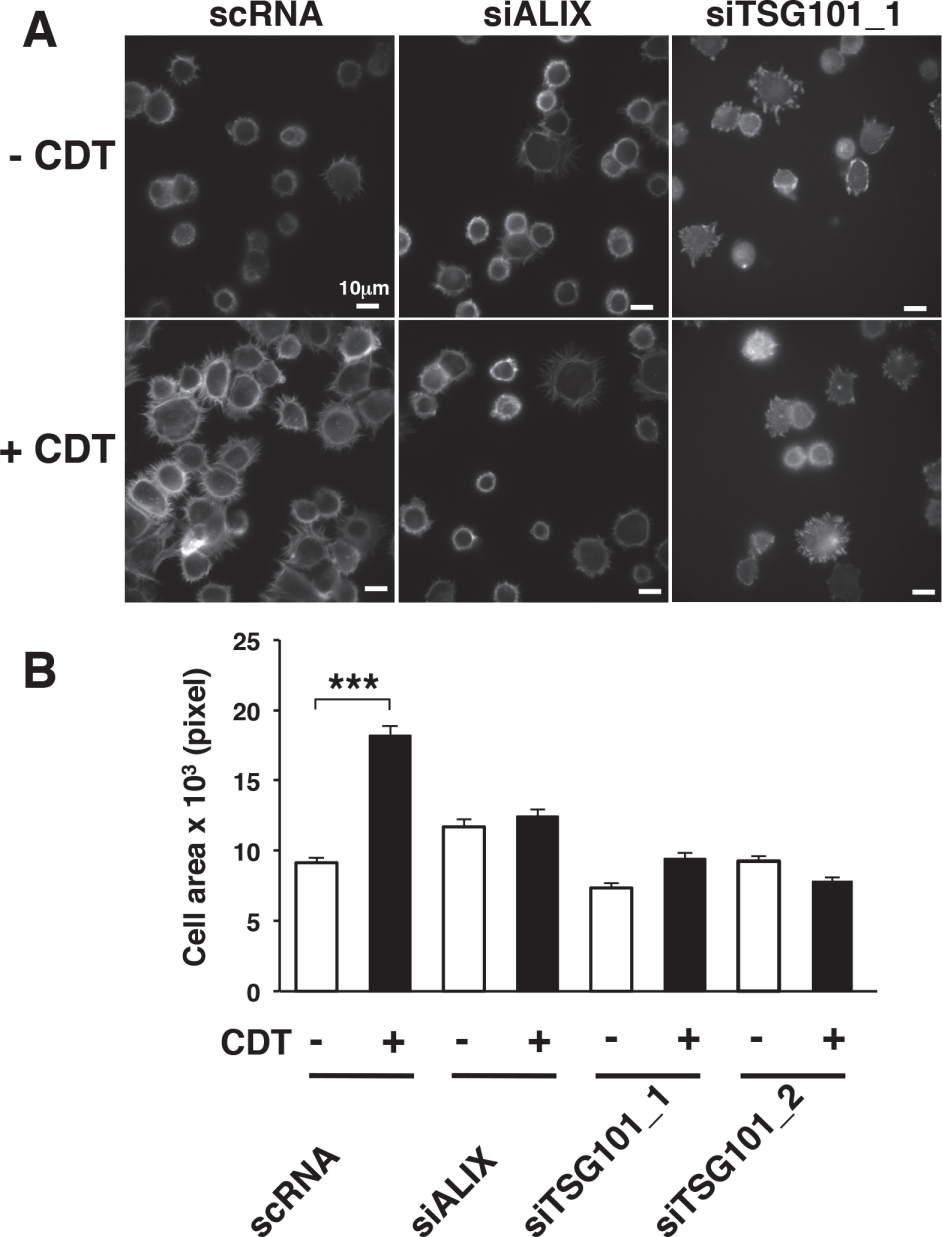
The ESCRT proteins TSG101 and ALIX are required to promote integrin β1-mediated cell spreading. **A.** HeLa cells transfected with the non-silencing siRNA (scRNA), the ALIX or TSG101 specific siRNAs (siALIX and siTSG101) were left untreated (CTR) or intoxicated with CDT (1μg/ml) for 6h, prior adhesion for 20 minutes on fibronectin-coated glasses. The figure shows representative micrographs where the actin cytoskeleton was visualized by phalloidin staining. **B.** Quantification of the cells area was performed as described in Fig 1. The data are presented as the mean ± SEM of three independent experiments. Statistical analysis was performed using the Student t-test. *** p value < 0.001.

Upon binding to their ligands, integrins are endocytosed and recycled back to the plasma membrane or transported via the ESCRT complex to the multivesicluar body (MBVs), for further trafficking to the lysosome (reviewed in [37]). This last step is essential for the regulation of cell spreading in fibroblasts [38]. Based on these observations, we investigated whether the ESCRT complex was required to transduce the integrin-mediated spreading observed in cells exposed to CDT. To this end, endogenous expression of the ESCRT components ALIX and TSG101 was knocked down by siRNA in HeLa cells, prior intoxication and cell seeding onto fibronectin-coated coverslips. siRNA transfection resulted in 80% and 60% down-regulation of ALIX and TSG101, respectively (S3B–S3C Figs). Knock-down of each of these proteins was associated with an inhibition of cell spreading on fibronectin coated coverslips (Fig 7A and 7B).

**Fig 8:**
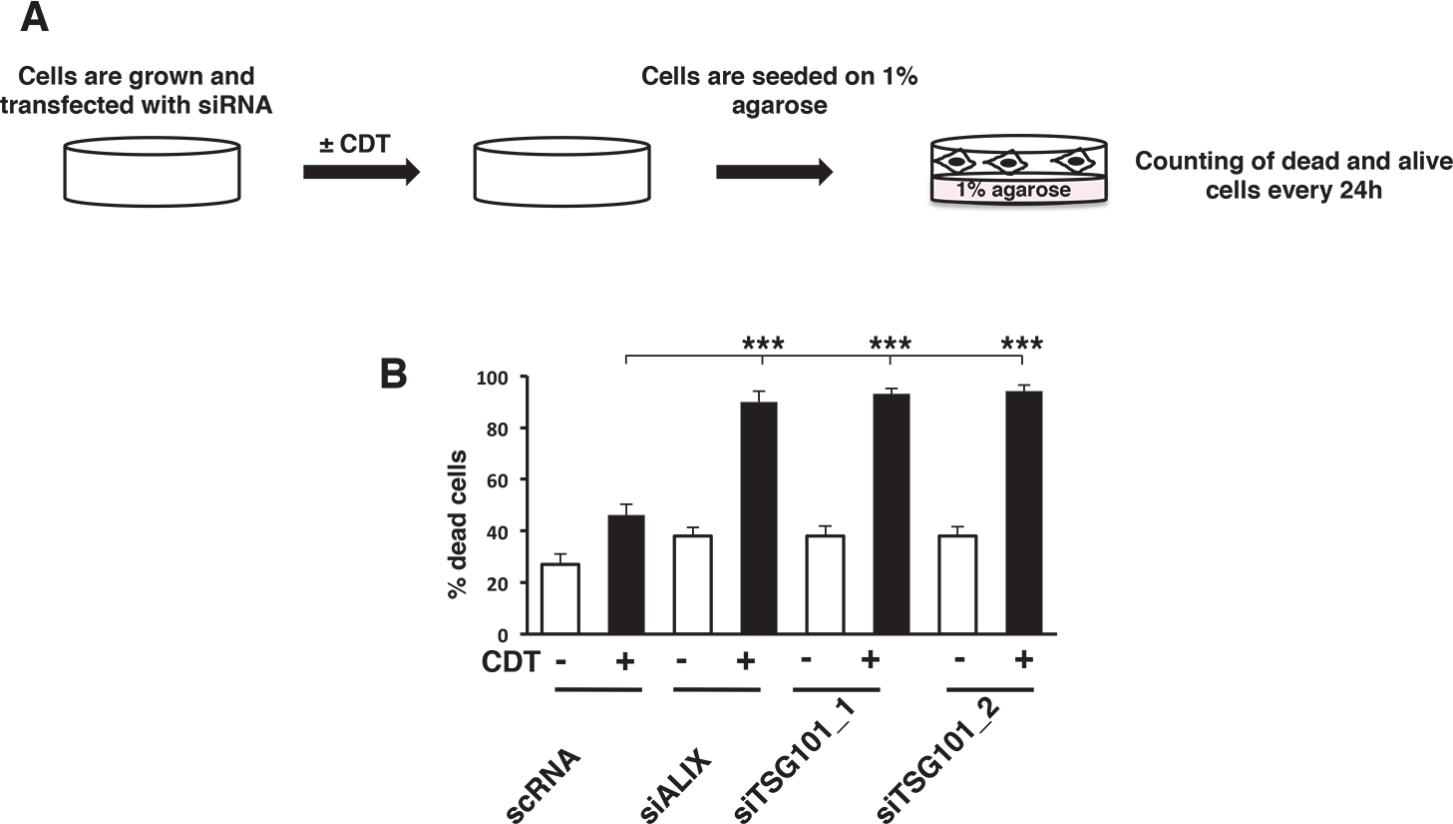
Integrin β1 signaling via the ESCRT proteins TSG101 and ALIX is required to promote anchorage independent cell survival. **A.** Scheme of the anchorage-independent cell survival assay. HeLa cells, transfected with the non-silencing siRNA (scRNA), the ALIX or TSG101 specific siRNAs (siALIX and siTSG101), were left untreated or intoxicated with CDT (1μg/mL) for 6h. Cells were detached with 2.5 mM EDTA in PBS, and seeded on 12 well plate pre-coated with 1% agarose in complete medium to prevent adhesion. Cell viability was assessed by Trypan Blue exclusion every 24h. **B.** Quantification of dead cells 96h after seeding on plate pre-coated with 1% agarose. The data are presented as the mean ± SEM of three independent experiments. Statistical analysis was performed using the Student t-test. *** p value < 0.001.

Sustained integrin activation is associated with enhanced cell survival in the absence of adhesion in several models [39,40]. Therefore we assessed whether enhanced integrin activation observed upon intoxication promoted adhesion-independent cell survival. To test this hypothesis, HeLa cells, transfected for 48h with siRNA specific for TSG101 or ALIX, or control siRNA, were left untreated or exposed to the toxin for 6h, detached with 2.5 mM EDTA and seeded onto wells pre-coated with 1% agarose to inhibit adhesion on the bottom of the well (Fig 8A). Cell viability was monitored by trypan blue exclusion in time kinetics experiments. No differences were observed at 48h and 72h after seeding, independently on the endogenous levels of TSG101 and ALIX and exposure to CDT (data not shown). However, a significant increase in cell death was detected in intoxicated cells with reduced levels of TSG101 or ALIX compared to that observed in cells transfected with the control siRNA and exposed to CDT for 96h (Fig 8B). Interestingly, the ESCRT protein TSG101 does not influence the adhesion-dependent cell survival [28], indicating that this component plays a role specifically on the regulation of cell survival in absence of adhesion.

**Fig 9:**
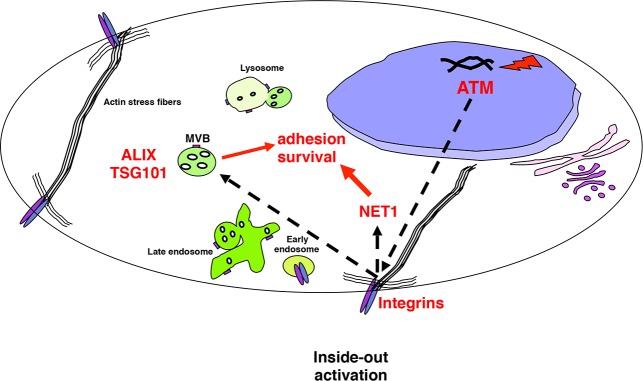
Summary of the study and significance. Genotoxin-induced DNA strand breaks activate the DNA damage response coordinated by the ATM kinase, which promotes the inside-out integrin β1 activation, leading to enhanced cell adhesion/spread, which is also dependent on the NET1-RhoA axis. The integrin response further relies on the ALIX and TSG101-dependent intracellular trafficking. The ESCRT-mediated integrin signaling is required to promote anchorage independent survival.

Collectively these data demonstrate that DNA damage induced by bacterial genotoxins promotes an ATM dependent inside-out activation of integrin β1. As results, intoxicated cells display enhanced spreading properties on fibronectin and capacity to survive in an anchorage independent manner. The transduction of the signal is dependent on the GEF NET1 and the ESCRT-mediated sorting complex (Fig 9).

Sustained integrin signaling in the presence of chronic intoxication with bacterial genotoxins may promote adhesion and anchorage independent survival favoring metastatic disease.

## Discussion

Integrins are membrane bound receptors that can control intracellular pathways regulating several processes, including cell survival and proliferation (reviewed in [25]). Our studies have focused on the cellular responses that promote integrin activation in cells exposed to bacterial genotoxins. We have identified a novel inside-out integrin signaling, which is regulated by the DNA sensor kinase ATM, leading to integrin β1 activation, cell spreading, and formation of FA. A key molecule in transducing the integrin signaling is the GEF NET1, and the response further requires the ESCRT-regulated integrin intracellular trafficking (Fig 9). In the context of chronic infection with genotoxin-producing bacteria these effects may favor survival of cells carrying genomic instability [18], leading to tumor initiation and/or progression and potentiate the metastatic properties of transformed cells.

Integrins play a pivotal role in the regulation of many processes that are de-regulated in cancer cells, such as motility, survival and proliferation. These receptors transduce extracellular cues bi-directionally across the plasma membrane, since they can be activated via an outside-in signaling triggered via interaction with the ECM, or an inside-out signal, which results in alteration of the integrin conformation, enhancing the affinity for the extracellular ligand [41].

The inside-out integrin activation has been mainly studied in the context of the cross-talk with membrane bound growth factor receptors or cytokine receptors. FGF- β, EGF and TGF- β treatment enhances the α1β1- and α2β1-mediated migration of hepatocellular carcinoma cell lines [42]. Activation of the EGF receptor promotes invasion via the Src-dependent phosphorylation of p130 CAS leading to αvβ5 integrin activation via the small GTPase Rap1 in a model of pancreatic carcinoma [43]. Similarly, activation of the Met receptor by HGF results in the tyrosine phosphorylation of integrin β4, promoting anchorage-independent growth [44]. Our study demonstrates that inside-out integrin β1 activation can also occur in response to events that start in the cell nucleus, such as induction of DNA damage.

Our data are not only relevant in the context of infections with genotoxin producing bacteria, but may contribute to understand the enhanced cell migration/invasion and proliferation that has been observed as a consequence of radiation therapy (reviewed in [45,46]). The integrin-dependent survival signals in irradiated cells is thought to be dependent on activation of receptor tyrosine kinases (RTKs), such as ERBB and IGF1R, which in turn can promote the inside-out integrin activation (reviewed in [46,47]). Some of these effects can be ascribed to the reactive oxygen species (ROS)-dependent inactivation of phosphatases, which lifts the inhibitory effect on RTKs activation (reviewed in [46]). However the molecular mechanisms governing these inside-out survival signals to ionizing radiation (IR) have not been fully elucidated. Our work contributes to identify some of these pathways (the ATM-dependent integrin activation), which are ROS-independent, since short-term intoxication with CDT does not induce oxidative stress [18].

Acquisition of metastatic potential by tumor cells is a multistep process, which requires invasion from the primary tumor site, intravasation, surviving the adverse conditions in the circulation flow, extravasation, and colonization of the distal site [48]. Integrins play a pivotal role, in many of these steps, and this can be achieved either by switching the pattern of integrin expression on the surface of tumor cells or by constitutive activation of the integrin dependent transduction pathway [48]. For example, cells of the buccal mucosa normally express αvβ5, while poorly differentiated squamous cell carcinomas showed a weaker staining for this integrin, and a concomitant increased expression of the integrin β6 [49]. Furthermore, constitutive activation of the transducers directly linked to integrin activation, such as the focal adhesion kinase (FAK) and the integrin-linked kinase (ILK), leads to anchorage-independent survival in several experimental set ups (reviewed in [50]). We have now demonstrated that intoxication with CDT or TT promotes integrin activation (Figs 1–4), specifically integrin β1 (Fig 4), and that interruption of this signaling compromises the ability of intoxicated cells to survive in absence of adhesion (Fig 8).

The enhanced activation of integrin β1 observed in our experiments is quite significant in the context of carcinogenesis, since this molecule has been shown to be up-regulated upon irradiation in breast cancer cells, leading to cellular resistance in response to IR treatment (reviewed in [51]) and promoting survival in 3D culture models [52]. Furthermore, over-expression of integrin β1 has been associated with disease progression in melanoma, ovarian carcinoma and non-small cell lung carcinoma (reviewed in [25]).

Surface integrins are internalized upon binding to their ligand and re-cycled back to the surface of the plasma membrane, and this step plays an important role in influencing their function (reviewed in [53]). Integrin recycling is mainly regulated by members of the Rab11 superfamily, which mediate the return to the plasma membrane via the Rab4-mediated short loop or the Rab11-dependent long recycling loop through the perinuclear region [53]. Several experimental evidences demonstrate an altered Rab-dependent integrin recycling in tumor promotion. For example, direct association of Rab25 with the integrin αβ1 promotes invasive migration in 3D models systems by directing the pool of integrin to the plasma membrane of the pseudopodal tips and the cell front [54]. Furthermore, altered recycling and constitutive activation of the same integrin heterodimer induced by expression of a mutant form of the tumor suppressor gene p53 promotes invasion and metastatic behavior in TERT-immortalized human retinal pigment epithelial cells [55]. Much less is known about the regulation of the integrin signaling via the trafficking through the ESCRT complex. Recent data demonstrate that the αβ1-mediated migratory capacity of fibroblasts requires endosomal sorting via MVB through the effector protein Hrs and the ESCRT1 TSG101 [56]. In the present study we have highlighted a key role of the ESCRT associated protein ALIX and TSG101 in mediating the activation of integrin-dependent signaling pathways (Fig 7), which are relevant for the acquisition of cell survival signals and anchorage independent growth (Fig 8), and may contribute to the acquisition of these pro-carcinogenic features in cells chronically exposed to CDT [18], as summarized in Fig 9.

### Conclusions

Our data identify a novel inside-out signaling pathway that leads to enhanced activation of integrin β1 and promotes several integrin-mediated functions, such as spreading and prevention of anchorage-independent cell death, in cells exposed to bacterial genotoxins. These data contribute to disclose novel molecular mechanisms promoting cell survival, and represent a step forward in understanding the transforming capacity of CDT [18,27].

## Supporting Information

S1 Fig Ionizing radiation promotes integrin β1 activation. HeLa cells were seeded and further left untreated (CTR, dotted line), irradiated with 8 Gy (IR, black line) and further incubated in complete medium for 6h, or intoxicated with CDT (1μg/ml) for 6h (grey line). Surface expression of the integrin β1 total levels (left panel) or its activate form (right panel) was assessed by FACS analysis as described in Material and Methods.
(TIF)

S2 Fig Effect of the KU-55933 inhibitor on the activation of the DNA damage response. HeLa cells were exposed to increasing concentration of the ATM inhibitor KU-55933 (ATMi) for 1h prior intoxication with CDT (1μg/ml). The levels of phosphorylation of the ATM substrates CHK2 and H2AX were assessed by Western blot analysis 6h post-intoxication.
(TIF)

S3 Fig siRNA knock down. HeLa cells were transfected with the non-silencing siRNA (scRNA), or the specific siRNA for NET1 (**A**), ALIX (**B**) or TSG101 (**C**). The levels of expression of the endogenous proteins were analyzed by western blot 48h after transfection. Actin expression was assayed as loading control. The figure shows a representative Western-blot analysis, and the quantification of three independent experiments (mean ± SEM). The data are presented as the ratio of the optical intensity (OD) of the specific band in cells transfected with the indicated siRNA and the optical intensity of the specific band in cells transfected with the control siRNA.
(TIF)
