# Supplementary figures and images for: Bacterial Genotoxins Promote Inside-Out Integrin β1 Activation, Formation of Focal Adhesion Complexes and Cell Spreading
Source: PLoS One. 2015 Apr 13;10(4):e0124119. doi: 10.1371/journal.pone.0124119 (PMC4395369; doi:10.1371/journal.pone.0124119)

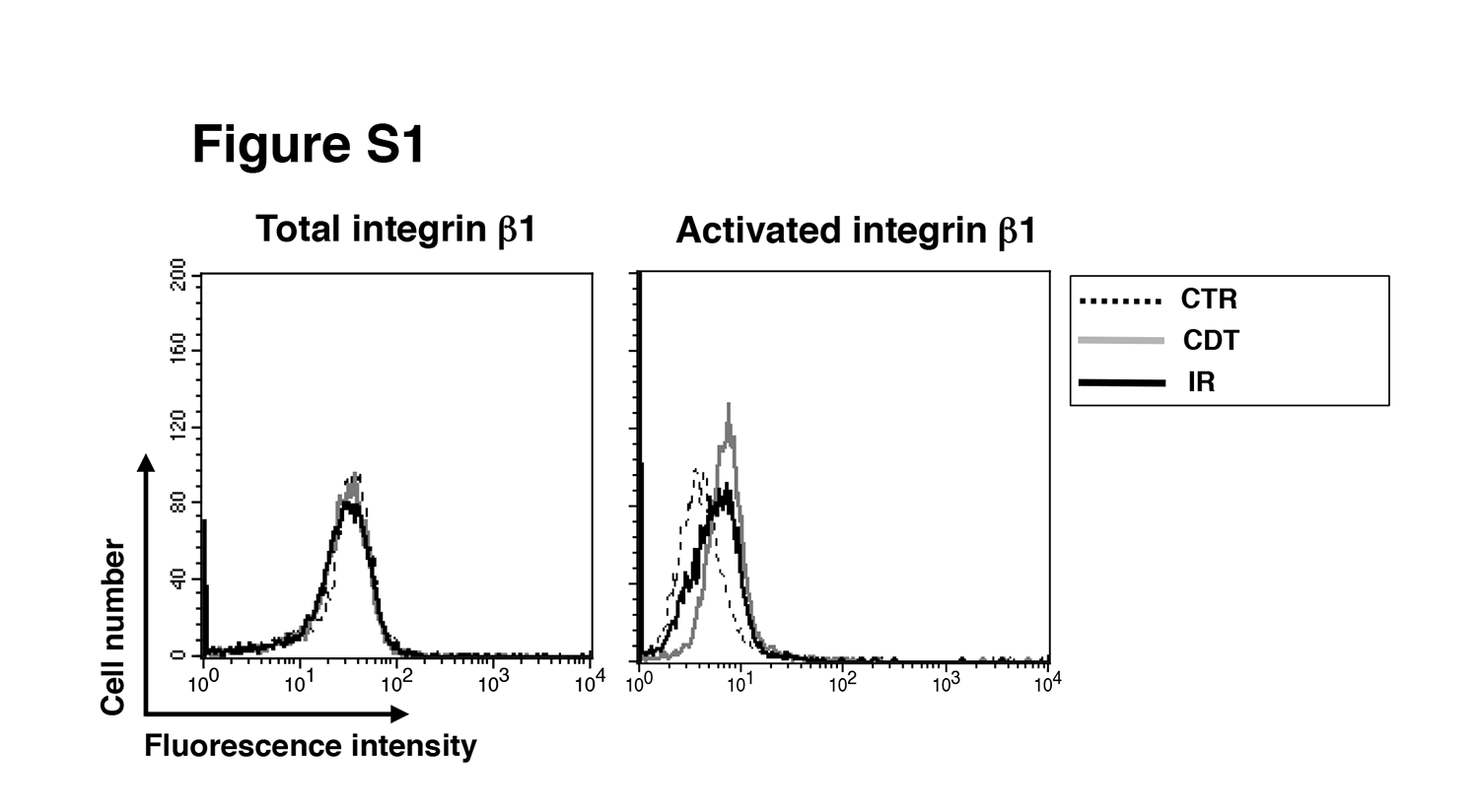

Supplement: S1 Fig — HeLa cells were seeded and further left untreated (CTR, dotted line), irradiated with 8 Gy (IR, black line) and further incubated in complete medium for 6h, or intoxicated with CDT (1μg/ml) for 6h (grey line). Surface expression of the integrin β1 total levels (left panel) or its activate form (right panel) was assessed by FACS analysis as described in Material and Methods. (TIF) [file pone.0124119.s001.tif]

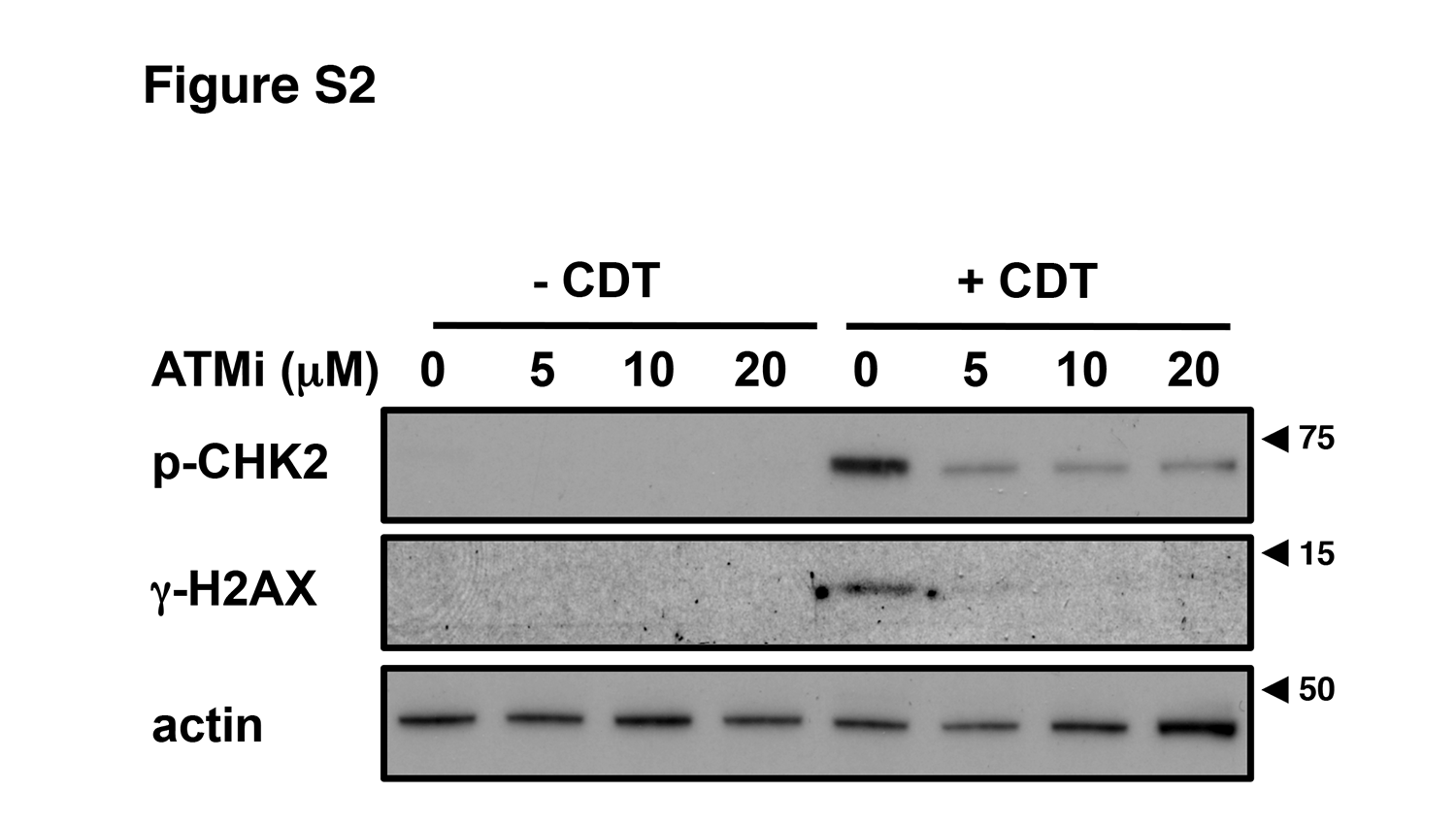

Supplement: S2 Fig — HeLa cells were exposed to increasing concentration of the ATM inhibitor KU-55933 (ATMi) for 1h prior intoxication with CDT (1μg/ml). The levels of phosphorylation of the ATM substrates CHK2 and H2AX were assessed by Western blot analysis 6h post-intoxication. (TIF) [file pone.0124119.s002.tif]

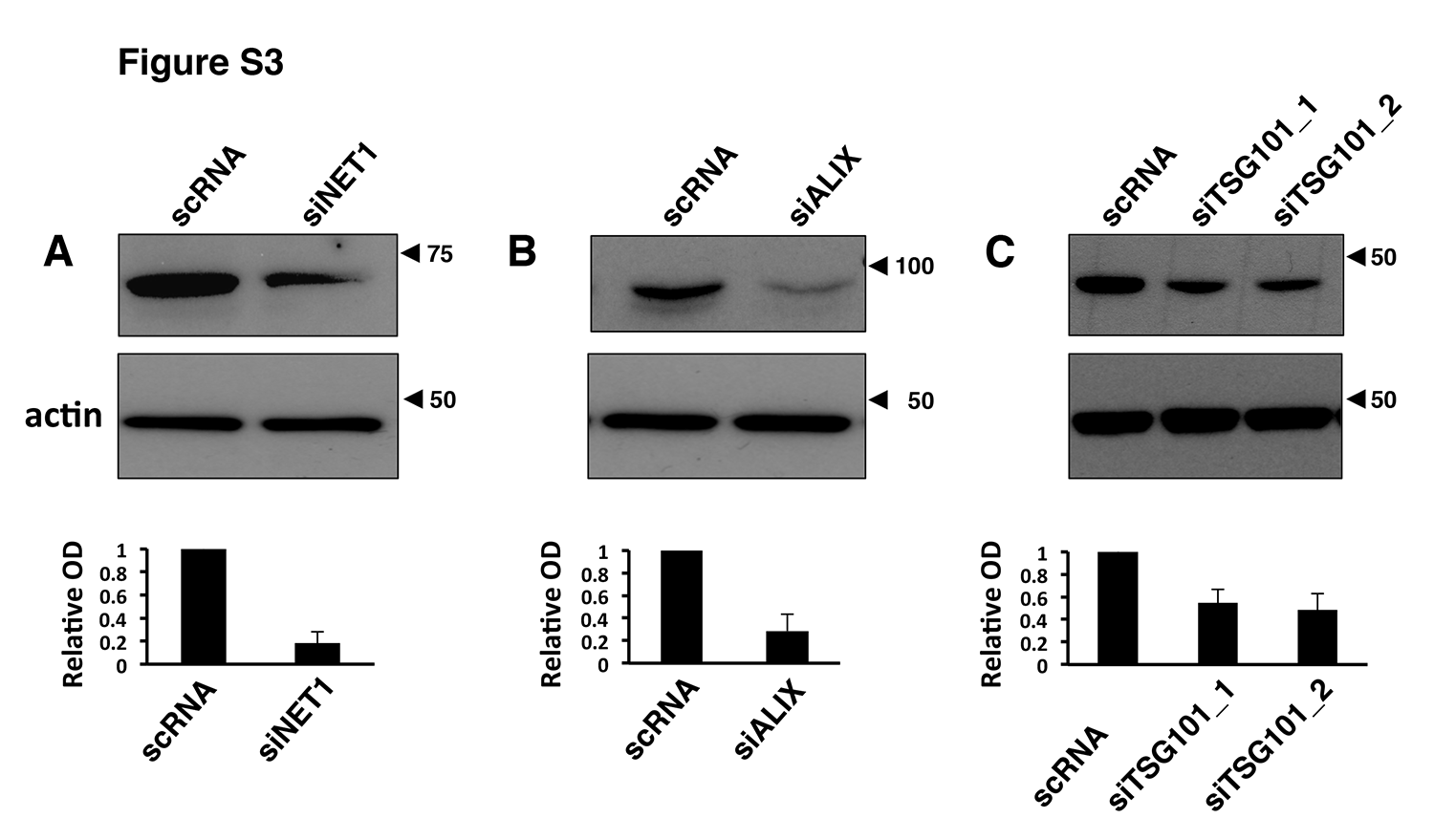

Supplement: S3 Fig — HeLa cells were transfected with the non-silencing siRNA (scRNA), or the specific siRNA for NET1 (A), ALIX (B) or TSG101 (C). The levels of expression of the endogenous proteins were analyzed by western blot 48h after transfection. Actin expression was assayed as loading control. The figure shows a representative Western-blot analysis, and the quantification of three independent experiments (mean ± SEM). The data are presented as the ratio of the optical intensity (OD) of the specific band in cells transfected with the indicated siRNA and the optical intensity of the specific band in cells transfected with the control siRNA. (TIF) [file pone.0124119.s003.tif]
